# Editorial: Causal inference in diet, nutrition and health outcomes

**DOI:** 10.3389/fnut.2023.1204695

**Published:** 2023-05-10

**Authors:** Qi Feng

**Affiliations:** Oxford Population Health, University of Oxford, Oxford, United Kingdom

**Keywords:** Mendelian randomization, nutritional epidemiology, causal inference, genetics, confounding

Causal inference in nutritional epidemiology is particularly difficult. Large randomized controlled trials, which are regarded as the gold standard, often have short periods of intervention and follow up, and focus on short-term outcomes, while evidences for long-term effects on hard clinical outcomes are sparse.

Observational nutritional epidemiology has long been faced with methodological difficulties, including complex confounding and dietary measurement error. It is known that individual diet is highly correlated with ethnicity, socioeconomic status (e.g., educational attainment, household income, occupation, etc.), health status, as well as other lifestyle factors (e.g., smoking, drinking, use of supplements and medication, etc.), all of which are in turn risk factors of health outcomes. Residual confounding remains even after adjusting for commonly measured confounders in regression models. A recent analysis has shown that residual confounding of socioeconomic status and lifestyle factors accounted for over 80% of the observational association between vegetable intake and cardiovascular disease (CVD) (1). Measurement of dietary exposure includes food consumption behavior and biomarkers. Biomarkers can be objectively measured, but not all food items or groups have validated and specific biomarkers (2); nevertheless, more progress is being made (3, 4). Data on food consumption behavior are commonly collected via self-report using 24-h food recall or food frequency questionnaires, especially in large cohorts.

Mendelian randomization (MR) studies are designed based on the accuracy of genotyping and the random assortment of alleles at meiosis, to minimize measurement error and mimic the randomization process in randomized controlled trials, thus improving the validity of causal inference. Therefore, MR can be considered as a potential alternative for causal inference in nutritional epidemiology (5, 6).

Nevertheless, concerns have been raised. MR relies on multiple assumptions, of which the most important is to identify plausible genetic instruments, and to serve as a valid proxy for the exposure of interest through its effects on either metabolic processes or consumption behavior. For biomarker-type exposures, finding genetic instruments is relatively easier, as we often have a degree of understanding of their metabolism. One of the typical examples would be serum vitamin D level (7). For diet intake behavior-type exposures, identifying valid genetic instruments is more difficult, unless there are specific key enzymes involved in the metabolic process; for example, *LCT* (lactase) and *ALDH2* (aldehyde dehydrogenase) genes are often used as instruments for intake of dairy products and alcoholic beverages, respectively. Overall, our knowledge is limited regarding how genes determine food intake behavior, which is also dependent on food accessibility, affordability, cultural background, and other environmental factors. As more large genome-wide association studies with fine mapping have been available, some mechanisms behind genes and dietary behaviors have been revealed. For example, olfactory receptors have been linked with fruit and vegetable intake (8), and lipid metabolism with meat-related diet (9). However, the functional effects of allelic variation for many other genes involved in dietary behavior traits remain unknown, which warrants further research.

In this Research Topic, we published four MR studies, two of which examined objectively measured biomarkers (serum iron and vitamin D), while the other two measured dietary behavior (tea drinking).

Wang et al. investigated serum iron related biomarkers and their associations with liver diseases. More specifically, they studied four iron status measures, including serum iron, transferrin saturation, ferritin and transferrin. For outcomes, they included five liver diseases (non-alcoholic fatty liver diseases, alcoholic liver disease, viral hepatitis, fibrosis/cirrhosis and liver cancer), and six liver injury biomarkers (alanine transaminase, alkaline phosphatase, aspartate aminotransferase, gamma-glutamyl transferase, direct and total bilirubin). They consistently found that liver injury biomarkers and liver diseases were positively associated with serum iron, transferrin saturation, and ferritin, but negatively with transferrin. Additionally, they also observed evidence for potential sex differences, with stronger associations in men than in women.

Liu et al. performed a wide-angled MR study by investigating the association between serum vitamin D level and 42 common diseases, covering cancers, respiratory diseases, cardiovascular diseases, chronic liver disease, cerebrovascular diseases, eye diseases and others. They found that vitamin D increased risk of cataracts in the Asian population, but reduced risk of non-senile cataracts in the European population.

Gao et al. and Sun et al. investigated the associations of tea intake with CVD and Alzheimer's disease, respectively. The genetic mechanism for tea intake may include taste and olfactory receptors, and caffeine metabolism as well as caffeine action targets. Guo et al. additionally found that tea intake was associated with lower risk of hypertension, heart failure and ischemic stroke, and hypertension mediated 17–24% of the associations with CVD. In Sun et al.'s study, they also investigated brain volume traits (total brain volume, gray matter, white matter, left and right hippocampus) and cerebral small vessel traits (mean diffusivity, fractional anisotropy, white matter hyperintensity volume, brain microbleeds, lobar microbleeds, and mixed type microbleeds). They observed evidence for a positive association between tea intake and Alzheimer's disease, and this may be mediated by decreased gray matter volume and hippocampus volume.

Concerns remain in applying MR in nutritional epidemiology, and once again, the most important and difficult part is to identify valid genetic instruments for dietary traits. To reinforce causal inference, we may need mediation analysis for mechanism exploration, dose-response analysis, to compare the findings to existing evidence, as well as always to be cautious when interpreting the results.

## Author contributions

QF wrote the first draft and critically revised it.
